# Fibrous dysplasia of bone associated with soft-tissue myxomas as well as an intra-osseous myxoma in a woman with Mazabraud's syndrome: a case report

**DOI:** 10.1186/1752-1947-5-239

**Published:** 2011-06-27

**Authors:** Wybren A van der Wal, Halil Ünal, Jacky WJ de Rooy, Uta Flucke, Rene PH Veth

**Affiliations:** 1 Department of Orthopaedic Surgery, Sint Maartenskliniek, Postbox 9011, NL-6500 GM, Nijmegen, The Netherlands; 2 Department of Orthopaedic Surgery, Rivierenland Hospital, Postbox 6024, NL-4000 HA, Tiel, The Netherlands; 3 Department of Radiology, Radboud University Nijmegen Medical Centre, Postbox 9101, NL-6500 HB Nijmegen, The Netherlands; 4 Department of Pathology, Radboud University Nijmegen Medical Centre, Postbox 9101, NL-6500 HB Nijmegen, The Netherlands; 5 Department of Orthopaedic Surgery, Radboud University Nijmegen Medical Centre, Postbox 9101, NL-6500 HB Nijmegen, The Netherlands

## Abstract

**Introduction:**

Mazabraud's syndrome is a rare but well-described disorder characterized by fibrous dysplasia in single or multiple bones associated with one or more soft-tissue myxomas. In this report, we describe what is, to the best of our knowledge, the first case involving an intra-osseous myxoma. This finding supports, and could provide new insight into, the pathological association between fibrous dysplasia and myxomas.

**Case presentation:**

In this report, we describe the case of a 49-year-old Caucasian woman known for years to have fibrous dysplasia in the left femur and tibia who presented with progressive pain in her left leg and soft swelling in the left quadriceps region. After surgical intervention with excision of the soft-tissue mass, the diagnosis of Mazabraud's syndrome was confirmed. During follow-up, our patient presented with a painless mass located on the lateral side of the left knee, next to a second, intra-osseous lesion with the same characteristics in the left lateral tibial plateau. The histopathological examination was consistent with a soft-tissue intra-osseous myxoma.

**Conclusion:**

In the international literature, 67 cases of Mazabraud's syndrome have been described so far.

To our knowledge, the present case report is the first to describe the combination of polyostotic fibrous dysplasia and intra-muscular as well as intra-osseous myxoma.

## Introduction

Mazabraud's syndrome is a rare but well-described disorder. It is characterized by fibrous dysplasia, which can develop in a single bone (monostotic) or in multiple bones (polyostotic), associated with one or more soft-tissue myxomas.

The first case was described by Henschen in 1926 [1]. A pattern of association between fibrous dysplasia and soft-tissue myxomas was described by Mazabraud *et al*. in 1967 [2].

Fibrous dysplasia is a benign, intra-medullary, fibro-osseous lesion and usually develops in childhood or early adult life. In the 67 cases of Mazabraud's syndrome described so far, most patients had the polyostotic form of fibrous dysplasia.

Soft-tissue myxomas are benign mesenchymal tumors. In Mazabraud's syndrome, they usually occur (1) when the patient is at a more advanced age and (2) in close vicinity of the bones most severely affected by fibrous dysplasia.

We present the first case of a patient with Mazabraud's syndrome with an intra-osseous myxoma next to soft-tissue myxomas.

## Case presentation

A 49-year-old Caucasian woman was referred to our center. She was known for years to have fibrous dysplasia in the left femur and tibia.

When she was 49 years old, an attempt was made to excise the fibrous dysplasia from the proximal femur. Extreme hemorrhaging complicated the operation. The attending orthopedic surgeon decided to refer the patient to our specialized center. At the time of her visit to our center, she complained about progressive pain in her left thigh and lower leg. Her clinical physical examination revealed soft swelling in the left quadriceps region.

**Figure 1:**
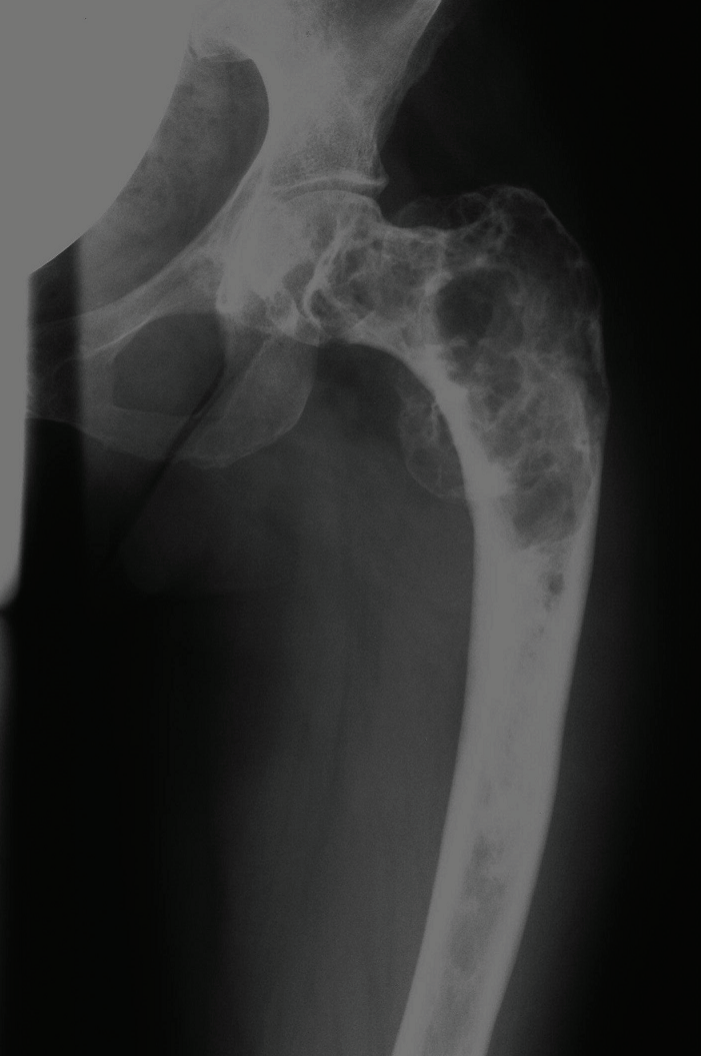
**X-ray of the left femur**. Anteroposterior plain film of the upper left leg with typical osseous changes consistent with fibrous dysplasia and shepherd's crook deformity.

**Figure 2:**
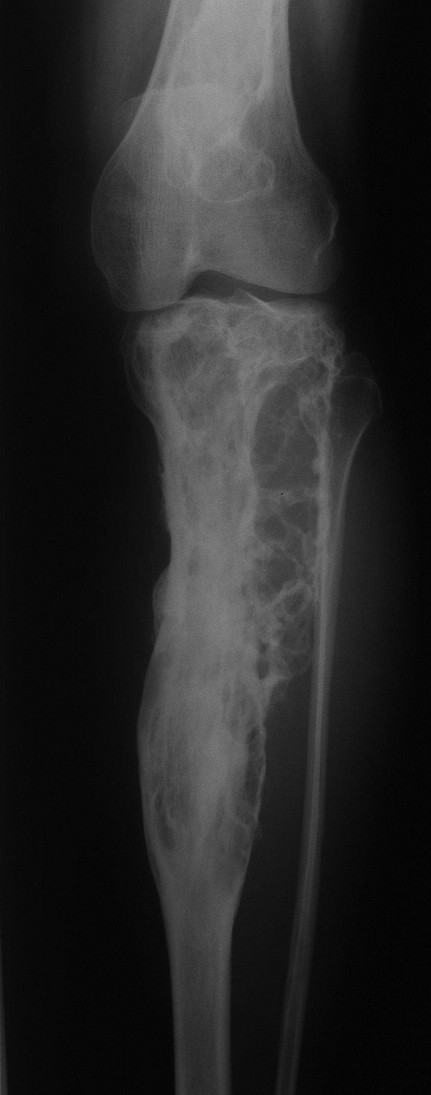
**X-ray of the left tibia**. Anteroposterior plain film of the left tibia showing fibrous dysplasia.

Conventional X-rays of the left leg showed typical features of fibrous dysplasia in the proximal femur (Figure 1) and the proximal tibia (Figure 2) with ground glass appearance and a shepherd's crook deformity.

Magnetic resonance imaging (MRI) of her left leg showed extensive fibrous dysplasia in the entire femur with expansive growth in the greater trochanter. The left proximal tibia also showed signs of fibrous dysplasia extending 20 cm distally from the tibial plateau. Four well-delineated, cyst-like, intra-muscular soft-tissue lesions were seen in the quadriceps region. Surgery was planned in two sessions. Prior to both operations, angiography and embolization of the pathologic vascularization in both the femur and the tibia were performed.

During the first operation, intra-lesional excision of the tumor of the left proximal femur was performed, followed by cryosurgery. A correctional osteotomy of the left hip was performed, followed by homologous bone transplantation and fixation with a proximal femoral nail. Because of a weakened femoral head, the femoral neck screw had to be stabilized with bone cement. Allograft bone chips were impacted to induce bone matrix. During the procedure, an incisional biopsy of the soft-tissue lesions in the quadriceps muscle was performed.

Three weeks later an intra-lesional excision of the fibrous dysplasia of the left tibia was performed, followed by cryosurgery and implantation of an massive allograft inlay in the tibia. The allograft was fixed with AO screws. During this procedure, the soft-tissue lesions in the quadriceps muscle were excised.

**Figure 3:**
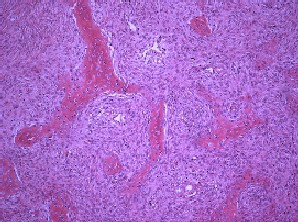
**Histology of fibrous dysplasia of our patient**. Photomicrograph showing curvilinear, slender trabeculae of woven bone surrounded by cellular fibroblastic tissue. There is no osteoblastic rim at the bone-stromal interface.

Microscopic examination of the bone lesions of both femur and tibia showed hypocellular fibrous tissue with irregular bone formations and without cytologic atypia compatible with fibrous dysplasia. In one lesion, the remnants of fracture callus were present (Figure 3).

**Figure 4:**
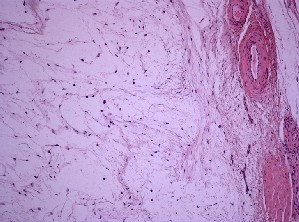
**Histology of myxoma of our patient**. Photomicrograph showing a paucicellular myxomatous lesion with dispersed, inconspicuous spindle cells.

The soft-tissue lesions showed a paucicellular tumor with spindle-shaped or stellate not atypical cells embedded in a loose myxoid alcian blue-positive inter-cellular matrix with sparse capillary blood vessels. Confocal microscopy revealed a thin fibrous capsule at the tumor margin; however, at the interface with skeletal muscle, infiltration between the individual muscle fibers was evident, which is typical of intra-muscular myxomas (Figure 4), proving the diagnosis of Mazabraud's syndrome in our patient.

Post-operatively, the patient's treatment consisted of non-weight-bearing mobilization for three months in plaster of Paris. During follow-up, plain films were obtained at six-week intervals. Eventually, these images showed consolidation of the femur osteotomy and incorporation of the allograft.

**Figure 5:**
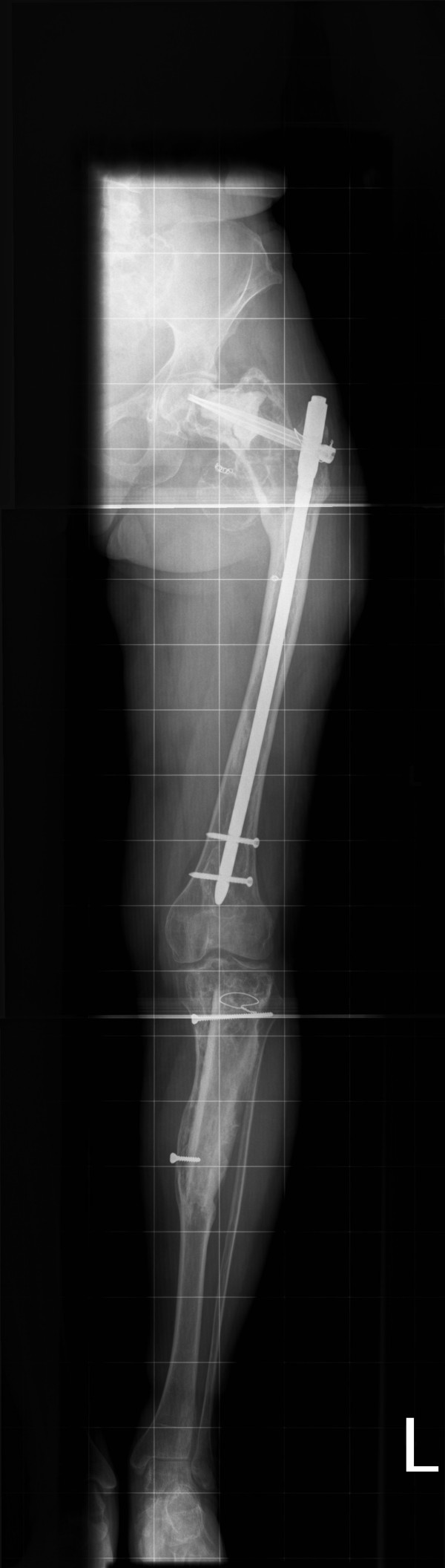
**Anteroposterior X-ray of the whole left leg**. Situation after fibrous dysplasia excision of the left proximal femur and left tibia with correctional osteotomy of the left hip and fixation with a proximal femoral nail, as well as allograft fixation with AO screws of the tibia.

**Figure 6:**
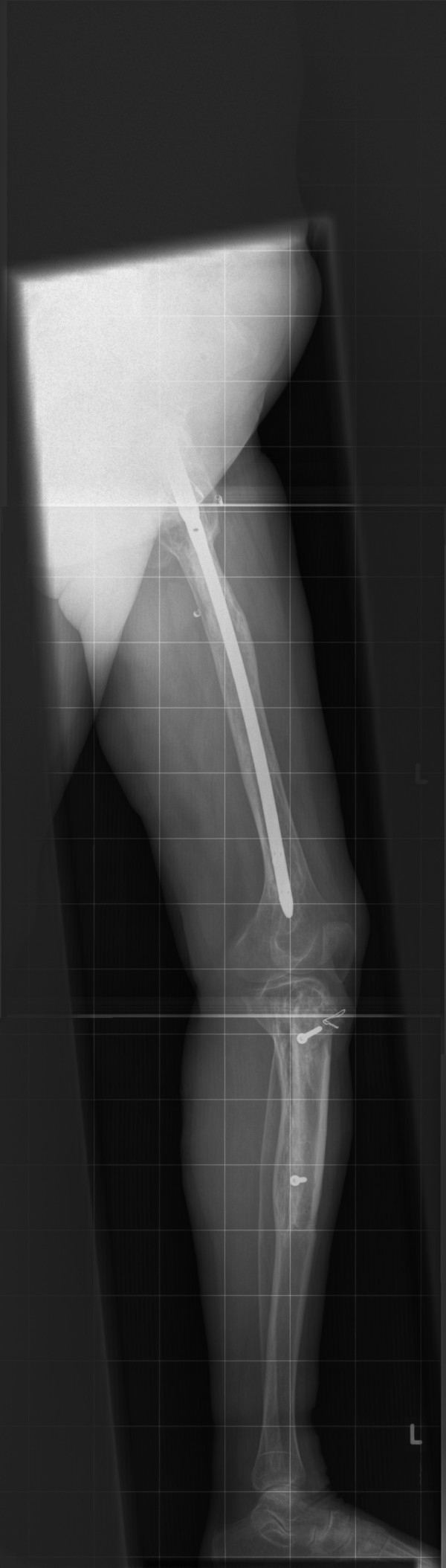
**Lateral X-ray of the whole left leg**. This X-ray shows the situation after fibrous dysplasia excision of the left proximal femur and left tibia.

Nine months post-operatively the patient developed progressive pain in the left thigh. Conventional X-rays showed protrusion of the femoral neck screw. Repositioning of the screw was performed. Her post-operative follow-up with X-rays obtained regularly (Figures 5 and 6) was satisfactory.

**Figure 7:**
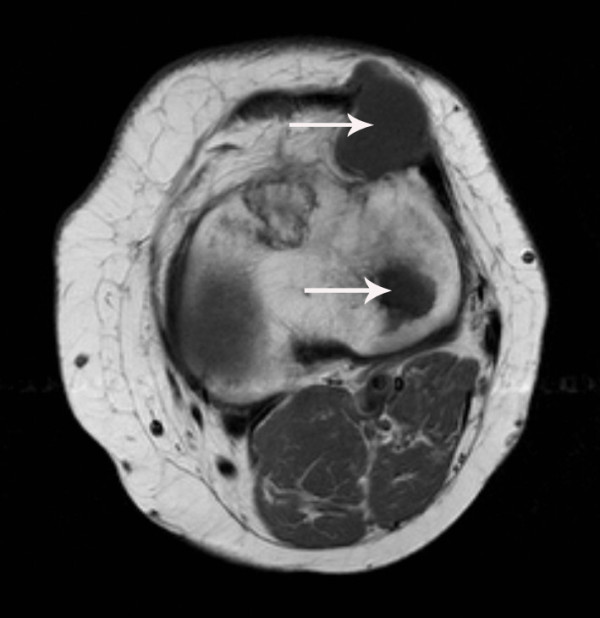
**T1-weighted magnetic resonance imaging scan of our patient**. Axial spin echo T1-weighted pre-contrast magnetic resonance imaging (MRI) scan of the left knee reveals two well-delineated masses (arrows) with a homogeneous low signal intensity in the lateral retinaculum and in the lateral tibia plateau.

**Figure 8:**
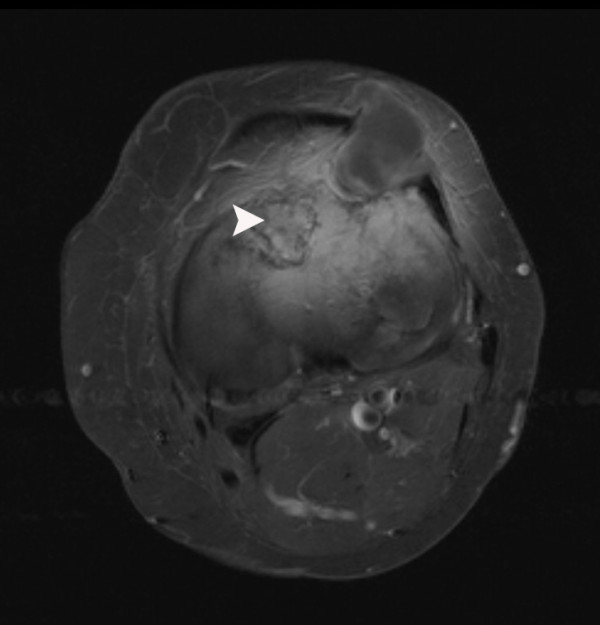
**T2-weighted MRI scan of our patient**. This T2-weighted image shows identical thin rim enhancement and heterogeneous intra-lesional enhancement in both masses. These findings may suggest myxomatous masses. In contrast, fibrous dysplasia can be noticed in the anterior tibial plateau (arrowhead).

Almost two years later the patient complained about a painless mass localized on the lateral side of the left knee. A solid mass was felt anterolaterally of the proximal tibia. Plain film radiographs did not show abnormalities; however, an ultrasound examination showed a homogeneous, hypoechogenic, soft-tissue mass with a cyst-like aspect. MRI showed a well-defined soft-tissue lesion in the lateral retinaculum and vastus lateralis muscle with a homogeneous high signal intensity on T2-weighted images and a homogeneous low signal intensity on T1-weighted images, consistent with fluid. A second intra-osseous lesion with an approximate diameter of 3 cm and the same signal characteristics and enhancement pattern was detected in the lateral tibial plateau (Figures 7 and 8).

**Figure 9:**
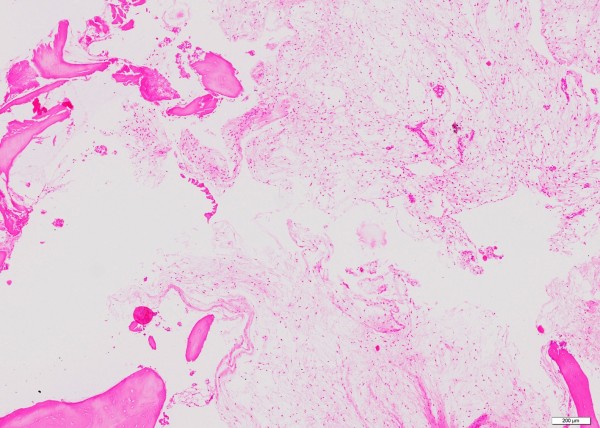
**Histology of intra-osseous myxoma of our patient**. Bone within a paucicellular myxoid lesion with small, bland spindle cells.

The lesions were considered to be a new soft-tissue myxoma and a relapse of fibrous dysplasia in the proximal tibia, respectively. Surgical excision of both lesions was performed without pre-operative biopsies, followed by cryosurgery and homologous bone implantation. A histopathological examination of the intra-muscular lesion showed a myxoma. As a unique finding, histopathological examination of the intra-osseous lesion in the lateral tibial plateau was consistent with myxoma as well (Figure 9).

During follow-up, MRI of the patient's left leg was performed at regular intervals. These scans showed no signs of recurrence in the first post-operative years.

Three years after her last operation multiple tumors in her left upper leg were felt, which raised clinical suspicions of myxomas. MRI scans showed five new soft-tissue lesions consistent with characteristics of myxoma in the upper and lower leg regions. All lesions were marked ultrasonographically and excised, followed by cryosurgery. Histopathological examination of all intra-muscular lesions confirmed the diagnoses. Post-operatively the patient recovered gradually. Every three months ultrasonography of her left leg was performed, which showed no signs of myxoma until 2009. By then, she had developed several new intra-muscular myxomas, which were not treated. At her last follow-up examination in early 2010, our patient was in good condition. Several myxomas had developed but were found to be stable on the basis of MRI.

## Discussion

Fibrous dysplasia in itself is not a rare disorder; it is reported to represent 5% to 7% of benign bone tumors [3]. Monostotic presentation is more frequent, and the lesions are thought to occur as a result of a developmental failure in the remodeling of primitive bone to mature lamellar bone and a failure of the bone to realign in response to mechanical stress. These features result in substantial loss of mechanical strength, leading to pain, deformity, and pathological fractures [4]. The etiology of fibrous dysplasia has been linked to a mutation in the G_s_α gene, leading to an increase in cyclic adenosine monophosphate, which leads to so-called downstream effects important in the pathogenesis of fibrous dysplasia [5].

An intra-muscular myxoma is usually a solitary lesion that is not generally associated with any other clinically apparent abnormalities [6]. It is a rare, benign mesenchymal tumor that is hypovascular, never exhibits an epithelial component, and probably never recurs locally [6].

Multiple intra-muscular myxomas are rare and usually are associated with fibrous dysplasia, a condition known as Mazabraud's syndrome. In Mazabraud's syndrome, the polyostotic form of fibrous dysplasia is present in the vast majority of patients. In general, the onset of fibrous dysplasia antedates the appearance of intra-muscular myxomas, and the soft-tissue lesions become apparent many years later, usually in the fifth or sixth decade of life [5]. The sites affected are predominantly the large muscles of the thigh, shoulder, buttocks, and upper arm. To our knowledge, no Mazabraud's syndrome case associated with an intra-osseous myxoma has been reported to date.

There are different hypotheses for the association between fibrous dysplasia and intra-muscular myxomas. Some authors have suggested a basic metabolic error in both tissues during the initial growth period [7]. Various authors have demonstrated that *GNAS1 *mutations exist in intra-muscular myxomas, which may play an important role in tumorigenesis in intra-muscular myxomas as well as in fibrous dysplasia [8].

The radiological diagnosis of fibrous dysplasia is generally established by plain films. Being larger, polyostotic lesions are more commonly accompanied by deformation, including coxa vara, the shepherd's crook deformity, bowing of the tibia, and protrusion acetabuli [4].

Ultrasound and MRI can be helpful to detect soft-tissue lesions in patients with Mazabraud's syndrome [9,10]. Both imaging modalities show circumscript, fluid-like lesions with a cystic or multi-cystic appearance. Therefore, recognition of Mazabraud's syndrome with its known association of fibrous dysplasia and benign soft-tissue myxomas is of utmost importance to establish the final radiology-based diagnosis. However, histopathological confirmation is always mandatory.

Histopathologically, the characteristics of fibrous dysplasia and intra-muscular myxoma are as described in this case report. Both lesions are completely benign, and malignant transformation in fibrous dysplasia is uncommon.

In many cases, treatment of fibrous dysplasia is not necessary, since the lesions are discovered incidentally on plain radiographs and are asymptomatic. Clinical observation through follow-up radiographs is warranted to verify that there is no progression.

Different studies have reported clinical improvement in patients with fibrous dysplasia after bisphosphonate therapy [11-13], although there is no histological evidence of abnormal osteoclastic activity in fibrous dysplasia of bone.

Surgical treatment of fibrous dysplasia may be necessary to correct a deformity, to prevent a pathological fracture, and/or to relieve pain in symptomatic lesions. Upper-extremity lesions can often be treated conservatively, but surgical intervention is required for many comparable lower-extremity lesions. Other factors influencing the type of intervention used are the size and biological behavior of the lesion and the patient's age.

The use of curettage only (with or without autogenous cancellous bone grafting) is associated with a high risk of recurrence, since the cavity or graft of normal bone is replaced gradually by dysplastic bone in the healing process, returning the patient to the pre-operative state. Cortical allografts last longer, since these grafts show far less and slower internal replacement by host bone and are therefore preferable for reconstruction.

The treatment of intra-muscular myxomas is dependent on the extent of the lesions, and these lesions should be excised if pain or pressure symptoms develop [10,14]. Our limited experience in using cryosurgery in soft-tissue tumors such as myxoma has shown that cryosurgery could be a powerful tool for the eradication of these tumors [15].

## Conclusion

Mazabraud's syndrome is a rare disorder; hence the small number of publications in the literature since it was first described by Mazabraud in 1967.

According to a recent article, there have been only 67 reported cases to date [16]. Our present report will probably be the 68th case, but it is the first report describing the combination of polyostotic fibrous dysplasia with intra-muscular and intra-osseous myxoma.

## Consent

Written informed consent was obtained from the patient for publication of this case report and any accompanying images. A copy of the written consent is available for review by the Editor-in-Chief of this journal.

## Competing interests

The authors declare that they have no competing interests.

## Authors' contributions

WAW did the literature research, studied the case, and composed the article. HÜ was a major contributor to the writing of the manuscript. JWJR reported the X-rays and MRI scans and contributed to the description of the radiological examinations. UF performed the histological examinations and contributed to the description of the pathological examinations. RPHV revised the manuscript critically. All authors read and approved the final manuscript.
